# Prevalence of dyslipidemia and the relationship between HbA1C and lipid profile in Moroccan patients with T2DM: a cross-sectional study

**DOI:** 10.11604/pamj.2022.43.86.35898

**Published:** 2022-10-19

**Authors:** Houda El Alami, Imane Haddou, Ghizlane Benaadi, Mustapha Lkhider, Lahcen Wakrim, Malika Allali, Omar Abidi, Hassan Ghazal, Najib Al Idrissi, Naima Nabih, Abderrahim Naamane, Abderrahmane Maaroufi, Naima Khlil, Salsabil Hamdi

**Affiliations:** 1 Research and Teaching Department, Environmental Health Laboratory, *Institut Pasteur du Maroc*, Casablanca, Morocco,; 2 Faculty of Medicine and Pharmacy, Laboratory of Chemistry, Biochemistry, Nutrition and Environment, University Hassan II, Casablanca, Morocco,; 3 Faculty of Sciences and Techniques of Mohammedia, Mohammedia, Morocco,; 4 Laboratory of Virology Unit, Immunovirology, *Institut Pasteur du Maroc*, Casablanca, Morocco,; 5 Laboratory of Human Molecular Genetics and Medical Genomics, Institut Supérieur des Professions Infirmières et Techniques de Santé (ISPITS) de Casablanca, Ministère de la Santé, Casablanca, Morocco,; 6 Department of Scientific, National Center for Scientific and Technical Research (CNRST), Rabat, Morocco,; 7 Department of Surgery, School of Medicine, Mohammed VI University of Health Sciences, Casablanca, Morocco,; 8 Ministry of Health, Chrifa Health Center, Casablanca, Morocco,; 9 Institut Pasteur du Maroc, Casablanca, Maroc

**Keywords:** Type 2 diabetes mellitus, dyslipidemia, HbA1C, cardiovascular complications

## Abstract

**Introduction:**

the increased prevalence of dyslipidemia in patients with type 2 diabetes mellitus (T2DM) results from uncontrolled hyperglycemia and consistently contributes to an elevated risk of cardiovascular complications. This study sought to estimate the prevalence of dyslipidemia and to investigate the relationship between glycated hemoglobin (HbA1C) and serum lipid levels in Moroccan patients with T2DM.

**Methods:**

a total of 505 patients with T2DM were included in this cross-sectional study, 77.4% with chronic complications and 22.6% without. The collected data were examined using statistical package for the social sciences (SPSS) version 20.0 software and appropriate statistical methods.

**Results:**

the data analysis showed that the mean and SD of age were 57.27±10.74 years. Among 505 patients with T2DM, the prevalence of hypercholesterolemia, hypertriglyceridemia, increased low-density lipoprotein cholesterol (LDL-C), and decreased HDL-C was 41.4%, 35.9%, 27.1%, and 17%, respectively. In addition, the data analysis showed that levels of total cholesterol (TC) (p≤0.001), triglycerides (p≤0.001), Low-density lipoprotein cholesterol (LDL-C) (p≤0.001), TC/HDL-C ratio (p=0.006), and LDL-C/HDL-C ratio (p=0.006) were significantly higher in T2DM patients with complications as compared to those without complications. The patients with HbA1C > 7.0% had significantly higher values of fasting blood glucose (FBG) (p≤0.001), total cholesterol (p≤0.001), triglycerides (p≤0.001), and TC/HDL-C ratio (p=0.025) as compared to the patients with HbA1C ≤ 7.0%. The HbA1C demonstrated a significant negative correlation with age (r=-0.139), and positive correlation with FBG (r=0.673), total cholesterol (r=0.189) and triglycerides (r=0.243).

**Conclusion:**

our results showed that HbA1C is the most important biomarker of long-term glycemic control and can also be a good indicator of the lipid profile.

## Introduction

Type 2 diabetes mellitus (T2DM) is a leading public health problem, that is affecting both developed and developing countries [1]. In 2016, diabetes mellitus affected 451 million people worldwide and was responsible for approximately 1.6 million deaths worldwide [2]. In Morocco, T2DM constitutes a major public health problem with a prevalence of 12.4% among adults [2]. Various chronic complications can be associated with diabetes mellitus, especially cardiovascular diseases, hypertension, stroke, nephropathy, retinopathy, neuropathy, lower limb amputations, and other metabolic disorders. In Morocco, the prevalence of chronic complications of T2DM was estimated to be 29.5% for retinopathy, 22.4% for cardiovascular diseases, 9.8% for nephropathy, 2.8% for diabetic foot, and 1.8% for neuropathy [3]. Dyslipidemia is defined as a group disorder of lipoprotein metabolism characterized by the presence of one or a combination of the following abnormalities: higher total cholesterol (TC), higher triglycerides (TG), and higher levels of low-density lipoprotein cholesterol (LDL-C) or reduced high-density lipoprotein cholesterol (HDL-C) levels [4]. It is very common among T2DM patients [5]. The interaction between dyslipidemia and hyperglycemia plays an important role in the onset and development of T2DM and its chronic complications [6]. In T2DM patients, dyslipidemia is considered a major risk factor for cardiovascular diseases, while hyperlipidemia enhances the risk of microvascular complications [7]. Many studies in different populations with different ethnicities, dietary patterns, and lifestyles found a significant variation in mean values for lipid parameters, confirming a strong correlation between glycated hemoglobin (HbA1C) and lipid parameters [8]. Glycated hemoglobin is commonly used for screening and diagnosis of diabetes, as well as for long-term monitoring of blood glucose levels in patients with T2DM [9]. Consistent with its function as an indicator of mean fasting blood glucose in patients with T2DM, the HbA1C predicts the risk of developing severe diabetes complications in patients with T2DM [10]. Moreover, the findings of the United Kingdom prospective diabetes study found that the risk of diabetes complications in patients with T2DM was strongly associated with uncontrolled hyperglycemia and that the control of hyperglycemia with a reduced level of HbA1C may be likely to diminish the risk of severe complications [10]. In addition to the traditional risk factors such as dyslipidemia, increased HbA1C has now been identified as an independent risk factor for stroke and coronary heart disease in patients with and without diabetes [8,11-13]. It is estimated that the risk of cardiovascular diseases increases by 18% for every 1% increase in absolute HbA1C levels in the diabetes population [8,14]. Khaw *et al*. have estimated that a reduction in the level of HbA1C by 0.2% can decrease mortality by 10% [15]. According to Vaag, improving glycemic control in patients with T2DM could be more important in preventing microvascular and macro vascular diseases than treating dyslipidemia [15]. This study aimed to determine the prevalence of dyslipidemia and to examine the effect of HbA1C on the lipid profile in Moroccan T2DM patients.

## Methods

**Study design:** this cross-sectional study was conducted between the periods of January 2017 and July 2018. The data for this study were gathered from T2DM patients aged-20 and up who were being followed in various health centers in Morocco's Casablanca-Settat region.

**Study population:** in total, we examined data from 505 patients with T2DM, which included 75 men and 430 women. The patients were recruited using these inclusion criteria: i) patients with T2DM who have been diagnosed using international criteria; ii) patients over the age of 20; and iii) patients´ agreement to give informed consent to participate in the study. We employed the following exclusion criteria: i) patients with type 1 diabetes or gestational diabetes; ii) patients under the age of 20; and iii) patients who had one outpatient visit or have incomplete data.

**Data collection:** participants were interviewed face-to-face by the study team, using a questionnaire to collect the necessary information, including sociodemographic data (gender, age, marital status, education, monthly income, social security), clinical data (family history of diabetes, diabetes duration, treatments, physical exercise, body mass index (BMI), and other diseases), and biological parameters. The BMI was categorized as under-weight (<18.5 kg/m^2^), normal-weight (18.5-24.9 kg/m^2^), overweight (25.5-29.9 kg/m^2^) and obese (>30.0 kg/m^2^). Glycemic control was categorized as good when HbA1C was ≤ 7%, and poor when HbA1C was >7% [16]. In this study, participants were defined as having dyslipidemia if they had one or more of the following conditions: TC >200 mg/dL), TG >150 mg/dL, LDL-C > 130 mg/dL), HDL-C < 40 mg/dL, or were taking antidyslipidemic drugs.

**Statistical analysis:** data were analyzed by using Statistical Package for Social Sciences (SPSS) version 20.0 for Windows. Categorical variables were presented as percentages, while quantitative variables were expressed as mean ± standard deviation (SD) or median (interquartile ranges). The student t-test or Mann-Whitney test was used to compare the means of continuous data (age, HbA1C, fasting blood glucose (FBG), total cholesterol (TC), triglycerides (TG), high-density lipoprotein-cholesterol (HDL-C), low-density lipoprotein-cholesterol (LDL-C), TC/HDL-C ratio, and LDL-C/HDL-C ratio. For the categorical variables, Pearson's X^2^tests and Fisher's exact tests were used to compare the percentages of the different parameters to determine if the difference was statistically significant or not. The Pearson correlation analysis and Spearman correlation analysis were applied to examine the different correlations. The P value of <5% was considered statistically significant.

**Ethical considerations:** this study was approved by the Ethics Committee of the Faculty of Medicine and Pharmacy in Rabat, Morocco (No. IORG0006594). All patients gave written consent after being informed of the objectives of the study.

## Results

**Characteristics of the participants:** among 505 subjects with T2DM, 85.1% were female and 14.9% were male. Our results show that the mean ± SD age of the participants was 57.27±10.74 years. The majority (63.3%) of participants are married and more than half are illiterate (58.6%). About 62.5% of subjects had a known family history of diabetes. In addition, 37.8% of subjects had a diabetes duration of more than 10 years, and 49.9% of them were taking oral antidiabetic agents. Approximately, 38.1% of the participants were overweight and 50.4% were obese. Most of the patients with T2DM were non-smokers (90.9%) (Table 1, Table 2). On the other hand, 77.4% of patients with T2DM suffered from at least one or more diagnosed chronic complications, while 22.6% had no recognized complications. Hypercholesterolemia, hypertriglyceridemia, decreased HDL-C, and increased LDL-C were found in 41.4%, 35.9%, 17%, and 27.1% of patients with T2DM, respectively (Table 2).

**Table 1:** socio-demographic characteristics of Moroccan patients with type 2 diabetes mellitus

Parameters	Categories	Number (%)
Gender	Females	430 (85.1)
	Males	75 (14.9)
Age (Years)	30-49	117 (23.8)
	50-59	162 (32.9)
	60 or above	213 (43.3)
Marital status	Single	32 (6.4)
	Married	317 (63.3)
	Widowed	100 (20)
	Divorced	52 (10.4)
Education	Illiterate	292 (58.6)
	Primary	105 (21.1)
	Secondary	56 (10)
	Higher secondary	28 (5.6)
	Graduate degree	23 (4.6)
Monthly income (in Dh)	<2500	342 (70.4)
	2500-8000	137 (28.2)
	>8000	7 (1.4)
Medical insurance	Without	93 (18.6)
	RAMED	306 (61.3)
	Insured	100 (20)
Smoking	Never smoked	459 (90.9)
	Currently smoking or Ex-smoker	46 (9.1)

**Table 2:** clinical and biological data among Moroccan patients with type 2 diabetes mellitus patients

Parameters	Categories	Number (%)
Family history of diabetes	Yes	308 (62.5)
	No	185 (37.5)
Duration of diabetes (yrs)	<5	148 (29.4)
	5-10	165 (32.8)
	>10	190 (37.8)
Diabetes treatment	Hygienic dietary rules only	46 (9.2)
	Oral hypoglycemic agents	249 (49.9)
	Insulin only	122 (24.4)
	Insulin + oral hypoglycemic agents	83 (16.6)
Regular exercise	Yes	241 (48)
	No	261(52)
Body weight	Under-weight	5 (1)
	Normal-weight	51 (10.5)
	Over-weight	185 (38.1)
	Obesity	245 (50.4)
Diabetes with complications	No	113 (22.6)
	Yes	388 (77.4)
HbA1c (%)	Good glycemic control (≤ 7)	120 (26.6)
	Poor glycemic control (>7)	331 (73.4)
Fasting blood glucose (mg/dl)	Normal	89(22.8)
	High	302(77.2)
Total cholesterol (mg/dl)	Normal (≤ 200)	189 (58.9)
	High (>200)	132 (41.4)
Triglycerides (mg/dl)	Normal (≤150)	216 (64.1)
	High (>150)	121 (35.9)
high-density lipoprotein-cholesterol (mg/dl)	Normal (≥ 40)	244 (83)
	Low (< 40)	50(17)
Low-density lipoprotein- cholesterol (mg/dl)	Normal (≤130)	213 (72.9)
	High (>130)	79 (27.1)
^2^Expressed in mean ± standard deviation		
^3^Expressed in median (interquartile range)		
*Statistically significant		

N, effective; OAD; oral antidiabetic agents; HbA1C, glycated hemoglobin; TC: total cholesterol; TG, triglycerides; HDL-C; high density lipoprotein cholesterol; LDL-C, low density lipoprotein cholesterol; RAMED, medical assistance scheme for the economically underprivileged

**Bio-clinical parameters of patients with T2DM in relation to T2DM complications:** data analysis showed that the risk of developing complications in T2DM increases significantly with advanced age (p≤0.001), longer duration of diabetes (p≤0.001), type of diabetes treatment (p=0.003), lack of regular exercise (p=0.034), and obesity (p=0.003). Furthermore, this risk was associated with high levels of TC (197 (170-235) vs 169 (141-194); p≤0.001), TG (132 (100-189) vs 91 (73-133); p≤ 0.001), LDL-C (112 (98-139) vs 99 (79-112); p≤0.001), TC/HDL-C ratio (3.95 (3.20-4.81) vs 3.49 (2.74-4.32); p=0.006), and LDL-C/HDL-C ratio (2.32 (1.87-2.90) vs 2.10 (1.34-2.54); p=0.006). In contrast, the median values of HbA1C (8.30 (7.0-10.40) vs 8.60 (7.10-11.65); p=0.102) and FBG (180 (134-245) vs 200 (135-256); p=0.516) were slightly lower in T2DM subjects with complications compared to those without complications, but the difference was not statistically significant (Table 3).

**Table 3:** bio-clinical parameters result of type 2 diabetes mellitus patients with or without complications

Parameter	Type 2 diabetes mellitus patients without complications (N=113)	Type 2 diabetes mellitus patients with complications (N=388)	p-value
Gender 1			
Female	9 (79.6%)	33 (86.9%)	0.057
Male	23 (20.4%)	51 (13.1%)	
Age 2	53.98 ±12.17	58.19 ± 10.09	≤0.001*
Family history of diabetes 1	65 (59.1%)	241 (63.3%)	0.427
Duration of diabetes			
< 5 years	57 (50.9%)	91 (23.5%)	≤0.001*
5-10 years	28 (25%)	135 (34.8%)	
> 10 years	27 (24.1%)	162 (41.8%)	
Diabetes treatment 2			
Hygienic dietary rules only	18 (16.1%)	28 (7.2%)	0.003*
Oral hypoglycemic agents	55 (50%)	193 (49.7%)	
Insulin only	28 (25.5%)	93(24%)	
Insulin + oral hypoglycemic agents	9 (8.2%)	74 (19.1%)	
Regular exercise 2			
Yes	68 (60.7%)	191 (49.4%)	0.034*
No	44 (39.3%)	196 (50.6%)	
Body weight 1			
Under-weight	2 (1.8%)	3 (0.8%)	0.003*
Normal-weight	19 (17%)	32 (8.6%)	
Over-weight	50 (44.6%)	133 (35.8%)	
Obesity	41 (36.6%)	204 (54.8%)	
Glycated hemoglobin (%) 3	8.60 (7.10-11.65)	8.30 (7.0-10.40)	0.102
Fasting blood glucose (mg/dl) 3	200 (135-256)	180 (134-245)	0.516
Total cholesterol (mg/dl) 3	169 (141-194)	197 (170-235)	≤0.001*
Triglycerides (mg/dl) 3	91 (73-133)	132 (100-189)	≤0.001*
High-density lipoprotein- cholesterol (mg/dl) 3	50 (39-58)	50 (43-57)	0.603
Low-density lipoprotein- cholesterol (mg/dl) 3	99 (79-112)	112 (98-139)	≤0.001*
Total cholesterol/high-density lipoprotein-cholesterol ratio3	3.49 (2.74-4.32)	3.95 (3.20-4.81)	0.006*
Low-density lipoprotein- cholesterol/high-density lipoprotein-cholesterol ratio 3	2.10 (1.34-2.54)	2.32 (1.87-2.90)	0.006*

1 Expressed in frequency (%)

2 Expressed in mean ± standard deviation

3 Expressed in median (interquartile range)

* Statistically significant

**The association of bioclinical and lipid parameters with HbA1C in T2DM patients:** the impact of glycemic control on various lipid parameters was evaluated by categorizing all the patients with T2DM into 2 groups as per their glycemic index: the first group consists of patients with good glycemic control (HbA1C value ≤ 7.0%), and the second group consists of patients with poor glycemic control (HbA1C value >7.0%) (Table 4). Glycemic control showed a statistically significant association with age (p≤0.001), and type of diabetes treatment (p≤0.001). Patients with poor glycemic control had a significantly higher value compared to those with good glycemic control of FBG (203 (159-261) vs 123 (104-145); p≤0.001), TC (196 (165-229) vs 176 (154-199); p≤0.001), TG (133 (98-197) vs 98 (80-130); p≤0.001), and TC/HDL-C ratio (3.97 (3.24-4.67) vs 3.42 (2.74-4.60); p=0.025). However, there were no significant differences in the median values of HDL-C (52 (43-56) vs 50 (42-58); p=0.802), LDL-C (106 (94-123) vs 111 (96-134); p=0.211), and LDL-C/HDL-C ratio (2.22 (1.71-2.83) vs 2.29 (1.88-2.87); p=0.345) between T2DM subjects with good glycemic control and those with poor glycemic control (Table 4).

**Table 4:** comparison of bio-clinical and lipid parameters between the good and poor glycemic control groups according to HbA1C value (*statistically significant)

Parameter Good glycemic control group	Type 2 diabetes mellitus patients (N=505)		
	Good glycemic control group	Poor glycemic control group	p-value
Age (years)2	60.73± 10.12	56.28± 10.38	≤0.001
Duration of diabetes (%)1			
<5 years	42 (35%)	84 (25.5%)	0.085
5-10 years	39 (32.5%)	106 (32.2%)	
>10 years	39 (32.5%)	139 (42.2%)	
Diabetes treatment1			
Hygienic dietary rules only	21 (17.5%)	10 (3.1%)	≤0.001*
Oral hypoglycemic agents	70 (58.3%)	153 (46.9%)	
Insulin only	17 (14.2%)	93 (28.5%)	
Insulin+Oral hypoglycemic agents	12 (10%)	70 (21.5%)	
Regular exercise1			
Yes	64 (53.8%)	163 (49.5%)	0.428
No	55 (46.2%)	166 (50.5%)	
Body-weight1			
Under-weight	1 (0.9%)	3 (0.9%)	0.575
Normal-weight	16 (13.8%)	30 (9.4%)	
Over-weight	40 (34.5%)	124 (39%)	
Obesity	59 (50.9%)	161 (50.6%)	
Fasting blood glucose (mg/dl)2	123 (104-145)	203 (159-261)	≤0.001
Total cholesterol (mg/dl)3	176 (154-199)	196 (165-229)	≤0.001
Triglycerides(mg/dl) 3	98 (80-130)	133 (98-197)	≤0.001
High-density lipoprotein cholesterol ratio (mg/dl)3	52 (43-56)	50 (42-58)	0.802
Low-density lipoprotein- cholesterol (mg/dl) 3	106 (94-123)	111 (96-134)	0.211
Total cholesterol/high-density lipoprotein-cholesterol ratio3	3.42 (2.74-4.60)	3.97 (3.24-4.67)	0.025
Low-density lipoprotein- cholesterol/high-density lipoprotein-cholesterol ratio3	2.22 (1.71-2.83)	2.29 (1.88-2.87)	0.345

1 Expressed in frequency (%)

2 Expressed in mean ± standard deviation

3 Expressed in median (interquartile range)

* Statistically significant

**Figure 1:**
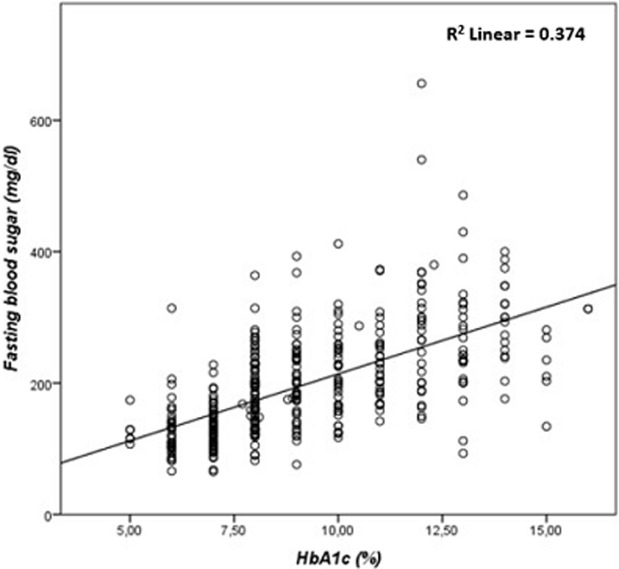
correlation between glycated hemoglobin and fasting blood sugar in patients with type 2 diabetes mellitus

**Figure 2:**
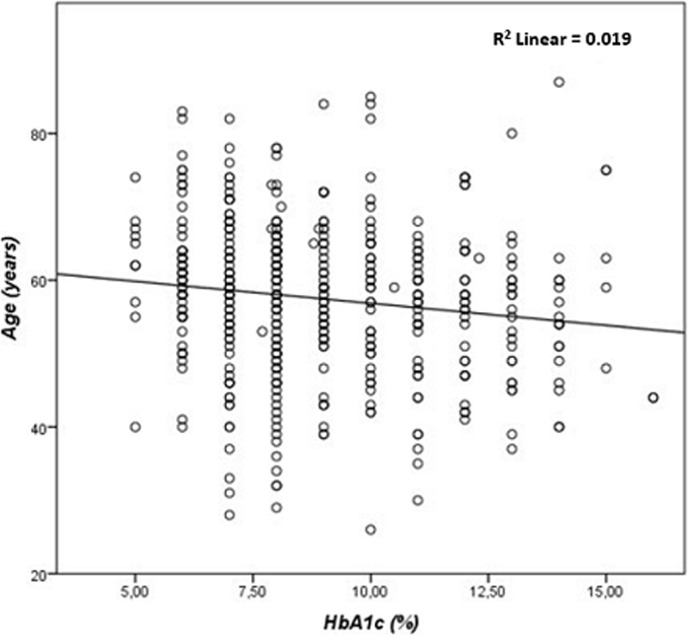
correlation between glycated hemoglobin and age in patients with type 2 diabetes mellitus

**Figure 3:**
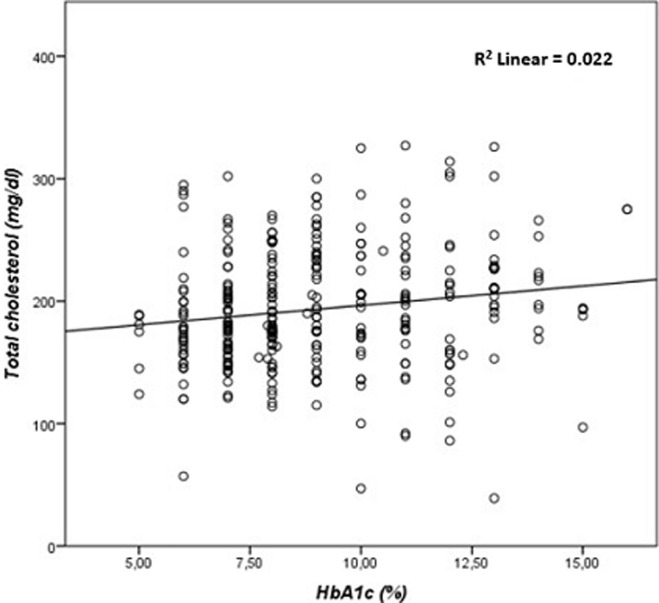
correlation analysis between glycated total cholesterol in patients with type 2 diabetes mellitus

**Figure 4:**
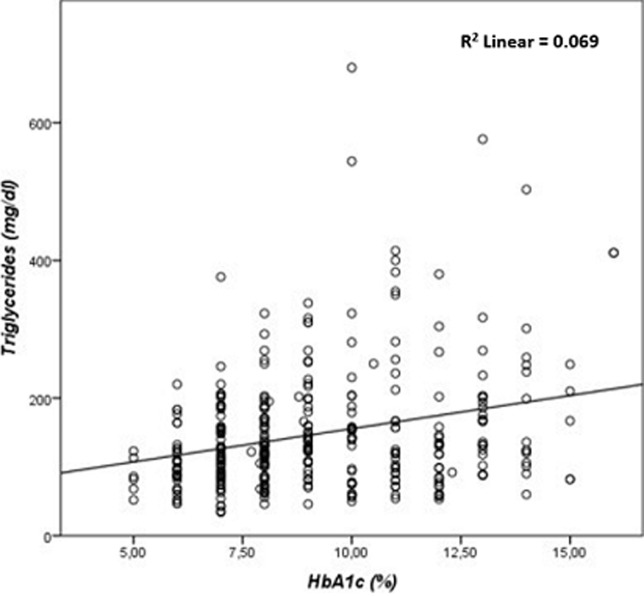
correlation between glycated hemoglobin and triglycerides in patients with type 2 diabetes mellitus

**Correlation between HbA1C, FBG, and lipid parameters:** the results of our study showed a highly significant correlation between HbA1C, and FBG (r= 0.673, p≤ 0.001) (Figure 1), TC (r=0.189, p ≤ 0.001) (Figure 2), and TG (r=0.243, p ≤ 0.001) (Figure 3). There was a significant negative correlation between HbA1C, and age (r= -0.139, p=0.004) (Figure 4). However, no significant correlation was found between HDL-C (r=0.028, p=0.636), LDL-C (r=0.104, p=0.081), TC/HDL-C ratio (r=0.121, p=0.057), and LDL-C/HDL-C ratio (r=0.062, p=0.312) and HbA1C (Table 5). The FBG was significantly and inversely correlated with age (r=-0.105, p=0.039), and directly correlated with TC (r =0.171, p=0.003), TG (r=0.198, p≤0.001) and LDL-C (r=0.143, p=0.019). Additionally, the TC demonstrated a significant correlation with TG (r=0.518, p≤0.001), HDL-C (r=0.172, p=0.005), LDL- C (r=0.656, p≤0.001), TC/HDL-C ratio (r=0.522, p≤0.001), and LDL-C/HDL-C ratio (r=0.359, p≤0.001). The TGs correlated positively with LDL-C (r=0.167, p=0.005), TC/HDL-C ratio (r=0.468, p≤0.001), and LDL-C/HDL-C (r=0.221, p≤0.001) (Table 5).

**Table 5:** correlation analysis between HbA1C, lipid parameters and fasting blood glucose

Parameter	HbA1C (%)		FBG (mg/dl)		TC (mg/dl)		TG (mg/dl)	
Correlation	Correlation	P-value	Correlation	P-value	Correlation	P-value	Correlation	P-value
Age	-0.139**	0.004	-0.105*	0.039	-0.070	0.217	-0.097	0.097
Glycated hemoglobin	1	-	0.673**	≤0.001	0.189**	≤0.001	0.243**	≤0.001
Fasting blood glucose	0.673**	≤0.001	1	-	0.171	0.003	0.198**	≤0.001
Total cholesterol	0.189**	≤0.001	0.171	0.003	1	-	0.518**	≤0.001
Triglycerides	0.243**	≤0.001	0.198**	≤0.001	0.518**	≤0.001	1	-
High-density lipoprotein cholesterol	0.028	0.636	-0.005	0.929	0.172**	0.005	-0.089	0.136
Low-density lipoprotein cholesterol	0.104	0.081	0.143	0.019	0.656**	≤0.001	0.167**	0.005
Total cholesterol/high-density lipoprotein cholesterol ratio	0.121	0.057	0.099	0.130	0.522**	≤0.001	0.468**	≤0.001
Low-density lipoprotein cholesterol/high-density lipoprotein cholesterol ratio	0.062	0.312	0.105	0.096	0.359**	≤0.001	0.221*	≤0.001

** Correlation is significant at the 0.01 level (2-tailed)

* Correlation is significant at the 0.05 level (2-tailed)

## Discussion

Diabetes is a multifactorial disease that results in a variety of lipid disorders. The relationship between diabetes and cardiovascular diseases is well-established and has been widely debated over the last few years. Both lipid profiles and diabetes mellitus have been shown to be strong predictors of metabolic disorders such as dyslipidemia, hypertension, cardiovascular diseases, and hyperinsulinemia. In this study, we aimed to determine the prevalence of dyslipidemia and to examine the association between HbA1C, lipid profile, and cardiovascular disease risk among Moroccan patients with T2DM. This cross-sectional study was conducted between January 2017 and July 2018 on 505 T2DM patients. Results showed a high prevalence of dyslipidemia (65.5%) among T2DM Moroccan patients. Fourty-one percent (41.4%) of patients had hypercholesterolemia, 35.9% had hypertriglyceridemia, 17% had a low HDL-C level, and 27.1% had a high LDL-C level, which are considered risk factors for cardiovascular diseases. Several studies, which agree with our findings, have found that TC, TG, and LDL-C levels are significantly higher in T2DM patients [17]. Goldberg demonstrated that insulin plays an important role in the production of apolipoproteins in the liver, which then regulates the enzymatic activity of lipoprotein lipase and cholesterol ester transport protein [18]. This mechanism could be responsible for dyslipidemia in T2DM [19]. In our study, we showed that 77.4% of patients with T2DM had one or more complications. This frequency is higher than that published by a previous Moroccan study (63.8%) [20] and some Arabic countries, such as Algeria (60%) [21], and Libya (68.7%) [22]. Like in other studies, our findings showed that age, longer duration of diabetes, therapy with a combination of OHA and insulin, absence of regular exercise, being overweight, and increased levels of TC, TG, LDL-C, TC/HDL-C ratio, and LDL-C/HDL-C ratio were the major factors associated with the development of diabetes complications in T2DM [23,24].

In our study, the correlation between HbA1C and the lipid profile in T2DM was examined. A highly significant positive correlation was revealed between HbA1C and FBG. These findings are similar to many other studies [25,17]. HbA1C also demonstrated a direct and significant positive correlation with TC and TG. Similar to our results, Mullugeta *et al*. reported that HbA1C was positively correlated with TC and high triglycerides in patients with T2DM [26]. This might contribute to predicting the TC and triglyceride status of patients with T2DM based on their degree of HbA1C and thereby identifying patients at higher risk of cardiovascular diseases. Lebovitz suggests the existence of a lipotoxic mechanism of triglycerides that interferes with gastric/neuronal mediated pathways that may regulate glycemic control in patients with T2DM [27]. Indeed, HbA1C levels were found to have a positive correlation with TC, LDL-C, and TAG in T2DM patients in a number of other studies. HbA1C has been defined by the Diabetes Complications and Control Trial (DCCT) to be a reliable glycemic control biomarker. Furthermore, an HbA1C value ≤ 7.0% was found to be appropriate to reduce the risk of cardiovascular complications [28]. Importantly, reducing HbA1C by 1% decreases the risk of myocardial infarction by 14%, the risk of microvascular complications by 37%, and the risk of diabetes-related mortality by 21%, underscoring the need to achieve and maintain HbA1C goals <7% for many adults [10]. On the other hand, increased physical activity and lifestyle changes have been shown to improve glycemic and dyslipidemia control. In the current study, patients with T2DM with poor glycemic control (HbA1C value ≤ 7.0%) experienced a significant increase in FBG, TC, TG, and TC/HDL-C ratio in comparison to patients with good glycemic control (HbA1c value ≤ 7.0%). Although many studies have proven the association between glycemic control (HbA1C) and lipid profiles in T2DM patients, the findings are fairly conflicting [26,29-31]. A study by Khan *et al*. on the variations in lipid profiles in 2220 T2DM patients found that lipid profile parameters for TC (5.49±0.04 vs 5.16±0.03 mmol/L), TG (2.13±0.04 vs 1. 88±0.02 mmol/L), HDL-C (1.1±0.01 vs. 1.21±0.08 mmol/L), and LDL-C (3.34±0.02 vs. 3.09±0.03 mmol/L) were increased in the group of patients with poor glycemic control [32]. These conflicting results could be explained by the relative stability of HbA1C. Indeed, HbA1C levels remain constant over a period of time, whereas lipid profiles and FBG levels change rapidly.

**Limitation:** the limitation of this study is the fact that if we wanted to estimate predictions, it would be necessary to follow-up patients since the prediction study is performed via a prospective study rather than a cross-sectional study. Therefore, a prospective study is needed to estimate this prediction. A combination of non-pharmacological therapies, such as medical dietary therapy, weight loss, and physical exercise, with pharmaceutical therapy is frequently required to improve lipid profiles in dyslipidemia. Finally, the findings of this study clearly demonstrate the importance of glycated hemoglobin in order to manage dyslipidemia and further reduce the risk of cardiovascular complications in patients with T2DM.

## Conclusion

The results of this study show a significant correlation between HbA1C and various circulating lipid parameters. Thus, we noted a significant difference in the lipid profile in two groups of HbA1C (good glycemic control and poor glycemic control). However, glycated hemoglobin could be used not only as a reliable biomarker of glycemic control but also as a good indicator for predicting dyslipidemia in patients with T2DM. Hence, an early diagnosis of dyslipidemia could be made with the glycemic parameter. Thus, continuous monitoring of blood glucose levels, HbA1C, and lipid profile is highly recommended in patients with T2DM in Morocco to reduce the risk of developing cardiovascular diseases.

### What is known about this topic

- The increasing prevalence of dyslipidemia in patients with type 2 diabetes mellitus suggests an urgent requirement for intervention to prevent macrovascular complications;
- In Morocco, there is a limited amount of research focused on the topic of dyslipidemia in type 2 diabetes mellitus patients.

### What this study adds

- This paper reports the prevalence of dyslipidemia in Moroccan patients with type 2 diabetes mellitus;
- This study has shown that HbA1C is associated with dyslipidemia in Moroccan type 2 diabetes mellitus patients;
- The results of this study can be confirmed that increased HbA1C represents a predictive factor for dyslipidemia.
